# Effect of two training modalities on sprint performance in young American football players

**DOI:** 10.3389/fspor.2025.1554055

**Published:** 2025-04-07

**Authors:** Valentin Prioul, Jean Slawinski, Steeve Guersent, Philippe Lopes, Pierre-Marie Leprêtre

**Affiliations:** ^1^ Univ Rouen Normandie, Normandie Univ, CETAPS UR 3882, Rouen, France; ^2^ Fédération Française de Football Américain, Pole de Performance, La-Plaine-Sain-Denis, France; ^3^ French Institute of Sport (INSEP), Laboratory Sport, Expertise and Performance (EA 7370), Paris, France; ^4^ Université d'Evry, Laboratoire de Biologie de l'Exercice pour la Performance et la Santé (LBEPS), Evry, France

**Keywords:** sprint acceleration, mechanical variables, undulating periodization, block training, team players, young talent evaluation, American football, performance

## Abstract

**Background:**

Time to perform 40-yard dash (40-yd) is a performance criterion in American football. Sprinting ability is strongly correlated with maximal values of horizontal power (PH_max_), Force (FH_0_) and Velocity (VH_0_). While numerous methods for developing sprint speed exist, few studies have focused on the effects of periodizations on the sprinting mechanical variables in young talented American football players.

**Objective:**

this study aimed to compare the effects of block (BP) and undulating (UP) training periodization modalities on 40-yard dash performance.

**Method:**

27 players from the Young French League of American football (17.1 ± 0.9 y, 179.9 ± 5.5 cm, 81.1 ± 14.9 kg) were randomly assigned in either the BP (*n* = 15) or UP (*n* = 12) group. Anthropometric characteristics, 40-yd performance, maximal velocity (V_max_), PH_max_, FH_0_ and VH_0_ were assessed before and after 10-wk intervention period.

**Results:**

Training resulted in the 40-yd performance increase of 3.72% (*p* < 0.001) and significant changes in V_max_ (+ = 6.13 ± 5.62%, *p* < 0.001) and VH_0_ values (+2.68 ± 4.14%, *p* = 0.004). BP intervention leaded higher improvements in time to perform 40-yd (4.45 ± 2.06 vs. 3.02 ± 1.93%, *p* < 0.001) and V_max_ (7.30 ± 6.63% vs. 4.54 ± 4.10%, *p* = 0.002,) compared to UP. No periodization effect was found in changes of VH_0_ (BP: 3.42 ± 4.31% vs. UP: 1.48 ± 3.88, *p* = 0.214).

**Conclusion:**

Our results showed that BP and UP were effective to increase sprint performance. Despite a similar training load, the block periodization of training had better effects on 40-yd performance compared to undulating training periodization in this population of talented young American football players.

## Introduction

American Football (AF) is characterized by the repetition of brief, highly intense actions. On average, play lasts 5.23 s and is interspersed with a recovery period of no more than 40 s, under penalty of the offensive squad (EO) (1). During the phases of play, players repeat numerous brutal accelerations and decelerations, enabling them to reach high speed values during sprints with (COD) or without (LD) change of direction (2). Two groups of authors have shown a relationship between sprint performance and the division in which the player played. Thus, the fastest athletes are those who play at the highest level (3, 4). An athlete's ability to accelerate forward has been linked to the capacity to produce and apply a high level of power to the ground in a horizontal direction, as well as a high level of external force at different speeds during sprinting. This ability is described by a linear relationship between force and speed (F-V) and a second-degree polynomial relationship linking power to speed (P-V). The relationships linking force and power to speed describe the change in maximum horizontal external force and power production as the speed of movement increases. These same relationships exist across mono and polyarticular movements. They provide an objective quantification of force/power production capabilities through the maximum power an athlete can develop in a horizontal direction (PH_max_), the theoretical maximum horizontal force an athlete can produce on the ground (FH_0_) and the theoretical maximum speed up to which the athlete is still capable of producing positive horizontal force (VH_0_) (5). The values of FH_0_ and VH_0_ are independent and associated with different physical and technical skills related to the production of a high level of horizontal force at low (FH_0_) and high (VH_0_) running speeds. These different mechanical variables are the result of a complex integration of different physiological, neural and biomechanical processes involving total external force production and characterize the different performance abilities of athletes (6, 7). Studies show that sprint performance variables, which are characterized by maximum speed (m.s^−1^), average speed (m.s^−1^) and distance covered over four seconds (m), are related to the efficiency of horizontal force application on the ground more than total force (8). These performance variables are strongly correlated with PH_max_, VH_0_ but not FH_0_ (8). Morin et al. (8) report that world-class sprinters have an F-V relationship more oriented towards VH_0_ than FH_0_. These results confirm the influence of maximum speed (V_max_) and athletes' ability to produce horizontal force on the ground at high speed on sprint performance (9). Through the templates present at the American football, the integration of body mass (BM) into the mechanical variables of horizontal force and power (FH_0_rel and PH_max_rel) increases the magnitude of differences between positions and/or categories (10). Together the previous findings suggested that players performing at the highest level are the fastest sprinters, and this performance has been correlated with VH_0_ and P_max_ (8, 9).

F-V profiles have been used in previous research as input for individualised training programs, as they help identifying an athlete's strengths and weaknesses. The results from the study of Devismes et al. (11) confirm the general hypothesis that sprinting skills increase with performance. As hypothesised, these authors (11) showed that F_0_, V_0_, P_max_ increase with the performance level in team sports. Periodization follows training planning and programming, with the aim of improving athletes’ sporting performance. Numerous authors have demonstrated the effectiveness of implementing training periodization for the development of strength, muscular power and improved sports performance (12–16). Handling variations in training loads (TL) and training cycles depend on the periodization implemented. There are several types of periodization in the literature, but we're only interested in the undulating (UP) and block (BP) models. The undulating model will lead to variations in performance by aiming to develop several physical qualities on a daily and/or weekly basis (17) whereas the block model will develop a physical quality over a period of two to four weeks (17). Some authors have sought to demonstrate the effectiveness of one periodization over the other in improving athletes' sports performance, but have failed to distinguish significantly which was preferable (14, 18–20). However, Painter et al. (19) would be in favour of BP, for a population of NCAA IA sprinters while two groups of authors (18, 20) tend towards a UP for a population with little or moderate experience. It is therefore difficult to make a choice based on the scientific literature, since the variables that make up the mesocycle and the muscle-strengthening program may be different (18).

Through knowledge of the mechanical variables associated with sprint performance, authors have highlighted the improvement in players’ ability to apply maximum horizontal force (FH_0_), PH_max_ and speed variables (i.e., V_max_ and VH_0_) following weighted sprint training (21–24). Consequently, this type of training would improve sprint performance over 30 m and intermediate times (22, 24, 25). The loads used for weighted sprints can be informed by a percentage of the body mass (22, 24) or by a percentage of the speed variables (i.e., V_max_ or VH_0_) (21, 23, 26). Improvement in the speed-oriented variables of the F-v profile (i.e., PH_max_, VH_0_ and V_max_) would move towards assisted (supra-maximal) or V_max_ sprints. Assisted sprint training sessions have been shown to increase average speed over the first fifteen yards in female college footballers (27). However, some authors show that training with unresisted or assisted sprints failed to improve mechanical variables training (21–24). Training methods for strength training are diverse and varied according to athletes’ goals, but intensities are generally prescribed by a percentage of 1-RM (%1-RM) (28). The use of a %1-RM may have some limitations, such as an inaccurate training load prescription (29). Indeed, athletes’ 1-RM is not fixed in time and can fluctuate according to various factors such as sleep, diet and fatigue caused by training. To overcome these limitations, some authors have turned to velocity-based training (VBT) to prescribe a load, number of sets and repetitions based on the speed of movement of an object (29–31). A linear relationship exists between speed and %1-RM, which can be used to prescribe exercise intensity. Moreover, with the accumulation of fatigue, speed decreases until it reaches the 1-RM speed, which can be used to avoid failure during the series and thus excessive fatigue (29–31). After learning about the factors associated with American football performance, this study was based on the development of physical qualities. For this reason, the objective was to compare the effects of two training periodization modalities on 40-yard dash performance. We hypothesized a beneficial effect of training on sprint performance, but did not know which periodization was more effective.

## Material and methods

### Population

Thirty-three American football players were selected to participate in the study. All of them joined the sports excellence program based on their results from the previous regular season and their success in the Combine test (32). Six players were excluded due to injury or participation below 80% of the training sessions. The distribution of final twenty-seven participants (17.1 ± 0.9 y, 179.9 ± 5.5 cm, 81.1 ± 14.9 kg, BMI: 25.1 ± 4.0 kg.m^−2^) and their anthropometric characteristics are presented in Table 1. This study was authorized by the Comité d'Ethique pour la Recherche en Sciences et Techniques des Activités Physiques et Sportives (CERSTAPS) and registered under IRB00012476-2022-16-02-154.

**Table 1:** Changes in anthropometric characteristics during the training period.

Variables	Group	Pre	Post	Δ%	*p* (training)	*p* (group)
Age	Block (*n* = 15)	16.5 ± 0.9				
	Undulating (*n* = 12)	17.6 ± 0.5				
	Total (*n* = 27)	17.0 ± 0.9				
Standing height (cm)	Block (*n* = 15)	180.6 ± 6.1	181.0 ± 6.1	0.2 ± 0.3	0.010*	0.606
	Undulating (*n* = 12)	179.1 ± 4.8	179.5 ± 4.9	0.2 ± 0.6	0.213	
	Total (*n* = 27)	179.9 ± 5.5	180.4 ± 5.5	0.2 ± 0.4	0.007*	
Body mass (kg)	Bloc (*n* = 15)	80.6 ± 16.6	81.3 ± 15.0	1.4 ± 4.2	0.409	0.354
	Undulating (*n* = 12)	81.9 ± 13.1	83.4 ± 14.7	1.8 ± 2.1	0.016*	
	Total (*n* = 27)	81.1 ± 14.9	82.2 ± 14.6	1.6 ± 3.3	0.050*	
Fat mass (%)	Block (*n* = 15)	14.8 ± 7.5	14.6 ± 6.7	5.1 ± 22.8	0.661	0.981
	Undulating (*n* = 12)	14.3 ± 5.8	14.3 ± 6.0	−1.1 ± 14.1	0.844	
	Total (*n* = 27)	14.6 ± 6.7	14.4 ± 6.3	2.3 ± 19.3	0.782	
Lean body mass (%)	Block(*n* = 15)	80.8 ± 7.1	80.6 ± 6.1	0.0 ± 1.7	0.838	0.678
	Undulating (*n* = 12)	81.7 ± 5.5	82.2 ± 5.9	0.6 ± 2.4	0.719	
	Total (*n* = 27)	81.2 ± 6.3	81.3 ± 6.0	0.3 ± 2.0	0.424	
BMI (kg/m^2^)	Bloc (*n* = 15)	24.7 ± 4.4	24.7 ± 3.9	0.6 ± 4.0	0.815	0.576
	Undulating (*n* = 12)	25.5 ± 3.5	25.9 ± 4.0	1.3 ± 2.2	0.079	
	Total (*n* = 27)	25.1 ± 4.0	25.3 ± 3.9	0.9 ± 3.3	0.235	

BMI, body mass index.

* Significant difference (*p* *<* *0.05*).

### Evaluation protocol

Following a six-week program of strength training in the form of circuit training and a four-week cycle of muscle hypertrophy using the cluster set method (76) all players completed evaluation tests. These tests were carried out over two non-consecutive days, separated by 48 h during which the players took part in low-intensity technical-tactical training sessions lasting less than two hours. For all tests, two trials were performed and only the best performance was retained (4). The same assessments were repeated at the end of the ten-week training period. All tests were supervised by the same operator.

### Evaluation of jumping performance and sprint strength-velocity profile

On the first day of evaluation, each athlete's standing height (SH) was measured using a portable height gauge (Leicester Tanita H001, Tanita corporation, Tokyo, Japan), and body mass (BM) and percentage fat mass (%) values using a professional impedance scale (Tanita DC360S, Tanita corporation, Tokyo, Japan). After a standardized warm-up, players performed a vertical rebound with free-arm countermovement (CMJ) (Optojump Next, Microgate, Bolzano, Italy) (33). Subsequently, the players performed a straight-line sprint of 40-yards or 36.6 m (i.e., 40-yd) on an uncovered synthetic pitch. Split times at 9.1 m, 18.3 m and 36.6 m were recorded using photocells (Witty System, Microgate, Bolzano, Italy). Position data were recorded as a function of time by a radar (Stalker ATS Pro II, Applied Concepts, TX, USA). This device was positioned 3 m directly behind the starting line and at a vertical height of 1 m to be approximately aligned with the athlete's center of mass (34). The variables of FH_0_, VH_0_ and PH_max_ were obtained through a method using speed data during straight-line sprinting (6). The speed curve over sprint time (VH[t]) was fitted by a mono-exponential function using least-squares regression:

(1) $$V(t)=V_{max}\times \;(1\;\text{–}\;e^{\text{–}t/\tau })$$

with *τ* the acceleration time constant. The horizontal acceleration of the center of mass can be expressed as a function of time, after deriving the velocity over time:

(2) $$a(t)=\frac{V_{\max}}{T}\times e^{\text{–}t/\tau }$$

The horizontal force (FH) was then modelled over time:

(3) $$F_{H}(t)=[m\times a(t)]+F_{air}(t)$$

with Fair as the aerodynamic friction force encountered during sprinting, which is calculated from the sprint speed and the estimated frontal body surface and a friction coefficient. Using the horizontal force and sprint speed values, we were able to determine an individual force-velocity relationship using least-squares linear regression. This relationship was then used to identify FH_0_ and VH_0_ as the x- and *y*-axis intercept. Maximum horizontal power was determined as:

(4) $$PH_{max}=\frac{{\mathrm{FH}}_{0}\;\times {\;\mathrm{VH}}_{0}}{4}$$

The relative variables (FH_0_rel, PH_max_rel) were determined by dividing the absolute value by the player's body mass. Samozino and al. (7) showed that sprint performance primarily depends on PH_max_ in football, rugby, basketball, and track and field. Depending on the distances to be covered, we can observe a dominance of force or velocity in the profiles. Additionally, Cahill and al. (21) used FH_0_ and PH_max_ as outcome measures in high school rugby and lacrosse players.

### Evaluation of the strength-speed profile of the lower limbs in the box squat

After a standardized 10-min warm-up including 5-min of mobility exercises (shoulders, hip and ankle) followed by a specific warm-up exercise for the lower limbs as unloaded lunge, hip thrust or squat, the players performed box squat (BX) movements in the presence of spotter, using an Olympic weightlifting bar equipped with an inertial measurement unit (V_max_pro®, EnodePro; Blaumann & Meyer—Sports Technology, UG, Magdeburg, Germany) (35). Briefly, athletes had to control the speed of movement during the descent phase and then hold the low position in contact with the box for around two seconds. At the instructor's signal, the player had to perform the ascent phase as quickly as possible. The initial load to be lifted was 20.0 kg for all participants. This was gradually increased by 20.0 kg until the mean propulsion velocity (MPV) fell below 1.00 m.s^−1^. From this speed, the load was incremented by 10.0 kg until the MPV dropped to 0.50 m.s^−1^, then by 2.5–5.0 kg until the series was stopped. Three attempts were made for each load with an MPV value greater than 1.00 m.s^−1^, two for loads inducing an MPV value between 0.65 and 1.00 m.s^−1^, and only one repetition for loads at which the MPV value fell below 0.65 m.s^−1^. Recovery time between each set was 4 min. Only the best repetition for each load was counted (best MPV) (36, 37). Bar displacement data were recorded at a frequency of 1,000 Hz and transmitted via a Bluetooth connection (65 Hz) to a tablet (38, 39). For each repetition, load, mean power (MP) and MPV values were collected in a spreadsheet (Microsoft® Excel version 16.16.27, Microsoft Corporation, Redmond, USA) according previous recommendations (36, 37). The force variable (F, expressed in N) was calculated as follows:

(5) $$F=\frac{MP}{MPV}$$

where MP is the average power, expressed in watts (w) and MPV, the average propulsion speed expressed in m.s^−1^.

The variable MP was calculated according to the equation proposed by Olovsson Ståhl and Öhrner (39):

(6) $$\mathrm{MP}\;=\mathrm{MPV}\times \left(g+\frac{\mathrm{MPV}}{t}\right)\times m$$

where g corresponds to the acceleration of gravity, i.e., 9.81 m.s^−1^, represents time in seconds and m, the mass of the load moved, expressed in kg.

Linear regression using the value of R^2^ to determine the percentage of variance was used when analyzing the load-velocity (L-V) and force-velocity-power (FVP) profiles of the box squat. If the R^2^ is close to 1, this means that the points are aligned on the right. Using the equation of a linear line, where a corresponds to the value of the theoretical maximum vertical velocity (Vv_0_) and b to the value of the theoretical maximum vertical force (Fv_0_), we were able to determine the maximum vertical power (PV_max_) which corresponds to the peak of the power-velocity relationship. The value of PV_max_ was calculated as follows:

(7) $$PV_{max}=\frac{FV_{0}\;\times VV_{0}}{4}$$

### Training protocol

The players were assigned to a training group (BP or UP) after randomization based on their 40-yard time. Then they completed a ten-week cycle of muscular power development using undulating or block programming according to the methodology of Painter et al. (19). Each week consisted of three sixty-minute strength training sessions, one sixty-minute sprint training session and three seventy-five- to one hundred and twenty-minute technical-tactical workouts. Table 2 shows the training schedule according to the development objective of the strength training sessions.

**Table 2:** Training schedule of team players according the undulating and block periodizations.

Group	Weeks 1–3	Weeks 4–7	Weeks 8–10
BP	*Core exercise (BX)*	*Core exercise (BX)*	*Core exercise (BX)*
	**Sessions 1, 2, 3 (Maximal strength)**	**Sessions 4, 5, 6, 7** **(Power)**	**Sessions 8, 9, 10** **(Maximal velocity)**
	Load: MPV 85% 1-RM	Load: 100% PV_max_	Load: Unloaded Olympic bar (20 kg)
	Repetitions: D15% MPV	Repetitions: D15% PV_max_	Repetitions: D5% MPV
	Sets: D15% MPV at first repetition or 25 repetitions.	Sets: Session 4–5 = 4 Session 6–7 = 5	Sets: 4
	Rest: 3 min	Rest: 2 min	Rest: 2 min
	*Complementary exercises (rest)*:	*Complementary exercises (rest)*	*Complementary exercises (rest)*
	**Sessions 1, 2, 3** 3 × 10 (2 min)	**Sessions 4, 5, 6, 7** 3 × 5 (2 min)	**Sessions 8, 9, 10** 3 × 5 (2 min)
UP	*Core exercise (BX)*	*Core exercise (BX)*	*Core exercise (BX)*
	**Sessions 1, 3** **(Power)**	**Session 4** **(Maximal strength)**	**Sessions 8, 9, 10** **(Maximal velocity)**
	Load: 10% PV_max_	Load: MPV 85% 1-RM	Load: Unloaded Olympic bar (20 kg)
	Repetitions: D15% PV_max_	Repetitions: D15% MPV	Repetitions: D5% MPV
	Sets: Session 4–5 = 4 Session 6–7 = 5	Sets: D15% MPV at first repetition or 25 repetitions.	Sets: 4
	Rest: 2 min	Rest: 3 min	Rest: 2 min
	**Session 2** **(Maximal strength)**	**Sessions 5, 6, 7** **(power)**	**Session 9** **(Maximal strength)**
	Load: MPV 85% 1-RM	Load: 100% PV_max_	Load: MPV 85% 1-RM
	Repetitions: D15% MPV	Repetitions: D15% PV_max_	Repetitions: D15% MPV
	Sets: D15% MPV at first repetition or 25 repetitions.	Sets: Session 4–5 = 4 Session 6–7 = 5	Sets: D15% MPV at first repetition or 25 repetitions.
	Rest: 3 min	Rest: 2 min	Rest: 3 min
		**Session 6** **(Maximal velocity)**	
		Load: Unloaded Olympic bar (20 kg)	
		Repetitions: D5% MPV	
		Sets: 4	
		Rest: 2 min	
	*Complementary exercises (rest)*	*Complementary exercises (rest)*	*Complementary exercises (rest)*
	**Session 1–3** 3 × 10 (2 min)	**Session 4** 3 × 10 (2 min)	**Sessions 8, 9, 10** 3 × 5 (2 min)
	**Session 2** 3 × 5 (2 min)	**Session 5–7** 3 × 5 (2 min)	**Session 9** 3 × 10 (2 min)
		**Session 6** 3 × 5 (2 min)	

BP and UP are block and undulating periodizations. BX, box squat; MPV, mean propulsive velocity; 1-RM, the percentage of one-repetition maximum; D15%, Deficit of 15% best mean propulsive velocity; D5%, Deficit of 5% best mean propulsive velocity; PV_max_, maximal vertical power.

The bold values indicate the aim for each session in the gym.

### Common features of weight training sessions

Of the three sessions, two were monitored using VBT, with bench press (DC) and BX as the main exercises. The other was dedicated to the shoulder press, following the recommendations of Hicks et al. (40), depending on the quality to be developed, and to individualized injury prevention exercises. Bench press and shoulder press data were not used in this study. To identify the loads associated with the physical quality to be developed during the sessions, we used C-v and power-velocity (P-V) profiles. Speed and percentage of 1-RM follow an almost perfect linear relationship, making it possible to identify the target speed at 85% 1-RM (31). The velocity associated with PV_max_ was used to identify the load through the C-V profile.

### Sprint features

All sprint sessions were directed by a percentage of maximum velocity (%V_max_) obtained during the sprint F-v profile assessment (21). Each athlete had a 10 m throw time, calculated according to V_max_ using the approach of Haugen et al. (41), which was recorded using photocells. All sprints were performed with a run-up phase before reaching the 10-m throw. Each session consisted of six sprints with a 4 min passive recovery. Only one specific sprint session per week was performed during the intervention.

### Characteristics specific to BX and sprint strength sessions

For the development of strength quality, the BX sessions were carried out using the MPV associated with 85% of the athlete's 1-RM. During the warm-up phase, if the athlete was 5% above the target speed, we increased the workload from 2.5 to 5.0 kg, and vice versa if the speed was reduced by 5%. When the athlete, in his series, reduced his work MPV by 15%, he had to stop and take three minutes of recovery. To end the BX exercise, the athlete had to either have the first repetition of his set at a MPV below 15% (i.e., 85% 1-RM) (42) or perform twenty-five repetitions in total (43). Strengthening exercises on the lower and upper limbs were performed following the BX by completing three sets of ten repetitions. For the sprints, the players had to perform six sled pushes over 10 m, with a 2.5 m run-up, in a time equivalent to 25% of V_max_ (21). This meant that an athlete with a V_max_ of 9.00 m.s^−1^ had to have a 10-m throw time of 4.44 s. A load on the carriage thrust was added corresponding to the desired split time. To ensure that the athlete remained within the range of maximum strength development, the split time was not to exceed 30% of V_max_.

### Characteristics specific to BX and sprint speed sessions

To develop the quality of speed, the BX was performed with an unloaded Olympic bar (i.e., 20.0 kg). The athlete's aim was to produce as much speed as possible during the concentric phase, by performing a jump to complete the extension. Due to the reduced fatigue caused by the absence of load, the athlete was stopped when the best MPV of the series decreased by 5%. The number of sets was set at four, with a passive recovery period of two minutes. Complementary assisted exercises (3 sets of 5 repetitions) were performed after the BX. For the development of sprint V_max_, a distance of 35-m was chosen by the recommendations of Rumpf et al. (44) and to approximate field conditions. A capture of the split time between the 25-m and 35-m was recorded and corresponded to 95%–100% of the athletes’ V_max_. Sessions were characterized by six unresisted repetitions with four to five minutes of recovery.

### Characteristics specific to BX and sprint power sessions

For maximum power development, BX was performed using an individual load to develop the PV_max_ calculated during the FVP profile assessment. The number of repetitions per set was set at a decrease of 15% of the day's PV_max_, with a passive recovery of 3 min between each set. The first two BX sessions consisted of four sets, followed by the last two with five sets. Following BX, plyometric exercises were performed in a horizontal and vertical plane over three sets of five repetitions with two minutes of passive recovery (40, 42). For the development of PH_max_ in sprinting, the load put on the sled corresponded to an optimal load (Lopt) on the recommendations of Cross et al. (26). To find out this load, we used 50% of the VH_0_ which gave us a speed and therefore a split time on the 10-m throw. The split time had to be no more than 55% or less than 45% of VH_0_. With this load the players performed six 10-m throw sprints, with 10-m run-up, followed by a four-minute passive recovery. The resistance load ranged from 30.0 to 162.5 kg for a mean relative load values of 1.5 ± 0.2 and 0.5 ± 0.1 kg.body mass^−1^ at 75% and 45% of V_max_, respectively.

### Programming

The number of training sessions of each quality was the same for both periodizations, as were the instructions for performing the main and complementary exercises. The warm-up before a sprint or strength training session was identical for both groups. Both periodizations consisted of two five-week mesocycles, with a two-week relief period in between.

For BP, the first mesocycle focused on maximum strength development for three weeks, characterized by loads ≥ 85% 1-RM in weight training and a 75% decrease in V_max_ in sprinting, followed by two weeks of maximum power development, characterized by Lopt during sprinting and weight training sessions. The second mesocycle aimed to develop maximum power and speed, characterized by two weeks at Lopt and three weeks at light load and non-weighted sprint. For UP, the intensity of BX and sprint training varied from week to week. The first mesocycle consisted of two strength and three power sessions in the weight room, and two strength, one power and two speed sessions in sprinting. The second mesocycle included one strength, one power and three speed sessions in the weight room and one strength, three power and one speed session in the sprint.

### Quantifying internal and external training loads

Training load (TL) was calculated by taking into account the number of sets, repetitions and intensity of the session (as a percentage of V_max_) and, for sprinting, the distance covered in metres was added (45). After each session, players were asked to give their feedback on the intensity (RPE) of the session, following the recommendations of Foster et al. (46). This intensity was then multiplied by the duration of the session to calculate the sRPE.

### Statistical analysis

All data were analyzed using JASP statistical software (version 0.17.2.1, JASP team, University of Amsterdam, Amsterdam, The Netherlands). Quantitative variables are presented as mean ± standard deviation. The distribution of the data was checked using the Shapiro–Wilk test, and the equality of variances of the data by the Levene test. The Student's *t*-test for independent samples was used to compare the percentage changes in 40-yd time and variables associated with sprint performance between the two training groups (block vs. undulating), and the Student's *t*-test for paired samples to observe the effect of the two periodizations on these same variables. Where necessary, the Mann–Whitney and Wilcoxon tests were used. The significance level was set at 5% (*p* < 0.05). A Spearman correlation was used to identify a correlation between variations in BM and sprint times (i.e., 40-yd; 20-yd; 10-yd). The use of G*Power software (version 3.1.9.6, University of Kiel, Germany) enabled us to estimate a necessary theoretical number of 21 players per group, i.e., a total population of 42 athletes. This estimate was based on the percentage variation in speed over 40-yd observed by Gavanda et al. (14), i.e., a calculated size effect (SE) value equal to 0.8, and on the application of a significance threshold set at 5% (*p* = 0.05) for statistical power of 80% (47). To minimize the bias associated with the difference between the number of subjects theoretically required and the actual number of participants, the ES value was calculated a posteriori. The ES was qualified as trivial if its numerical value was less than 0.2, low if between 0.2 and 0.49, medium if between 0.5 and 0.79 or large if greater than 0.8.

## Results

**Figure 1:**
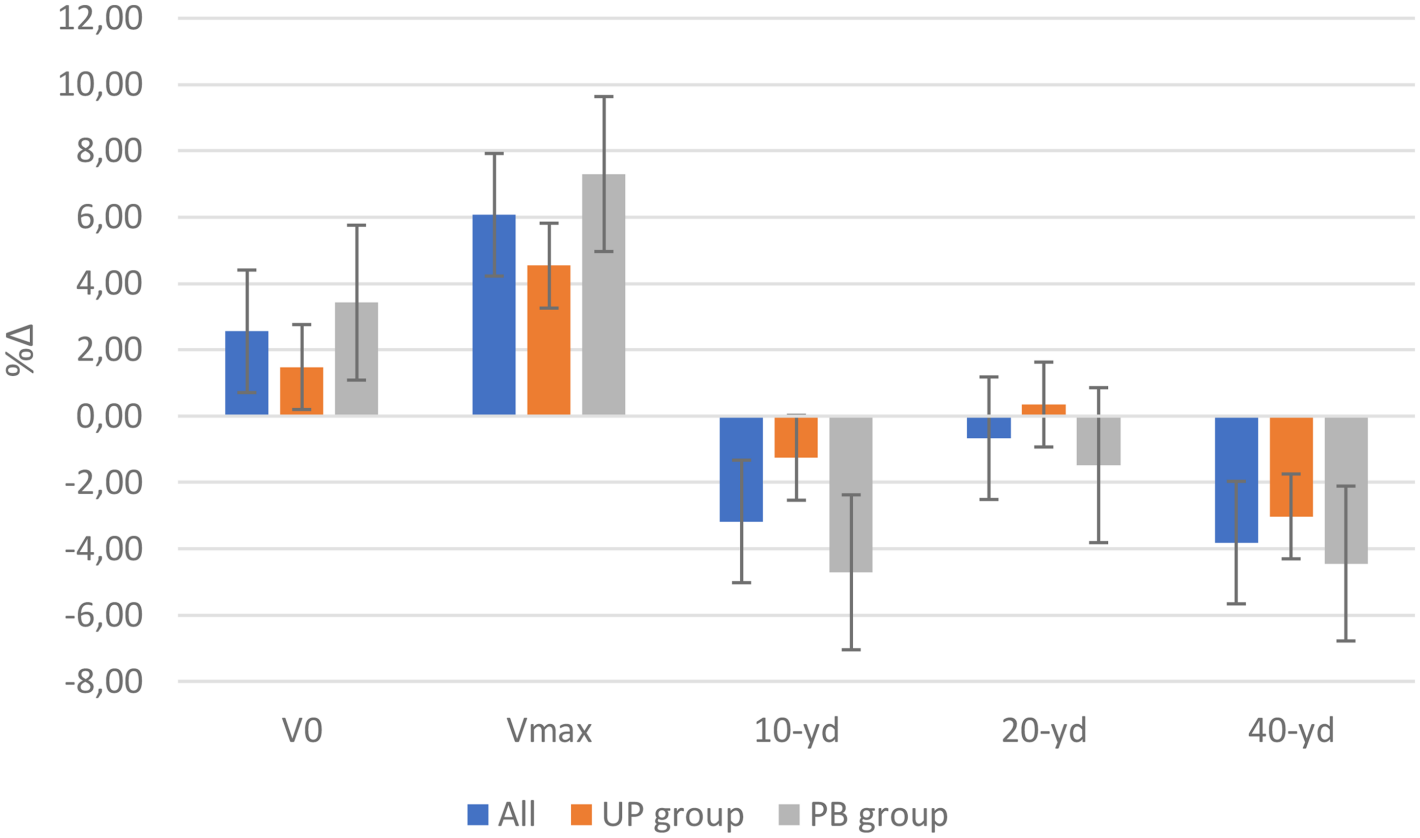
Effects of training programs on sprint performance. Legend: V_0_, V_max_ are theoretical maximal velocity and maximal speed. 10-yd, 20-yd and 40-yd presented the covered sprint distance expressed in yards.

The results of the anthropometric characteristics were presented in Table 1. No significant differences were observed for measurements taken at the beginning of the intervention. There was a time effect on SH and BM values. No significant differences were found between groups. The Student's or Wilcoxon test showed a significant difference on SH and moderate ES following the intervention for the whole sample (*p* = 0.007; ES = −0.54) and BP (*p* = 0.01; ES = −0.77). A significant increase in BM was found for the population as a whole (*p* = 0.05, ES = −0.40) and for athletes in the UP group (*p* = 0.016, ES = −0.77). All other anthropometric characteristics remained significantly unchanged, and ES was considered low to trivial except for BMI in the UP group (*p* = 0.079, ES = −0.56). No correlation was found between variations in BM or lean body mass and time to 40-yd. All measurements of sprint parameters for both training modalities were reported in Table 3. No significant differences were observed in the measurements taken at the start of the intervention. Figure 1 showed a significant improvement of 3.72% at 40-yd (*p* < 0.01, ES = 1.77). This improvement on the 40-yd was more marked for athletes in the BP group (4.45 ± 2.06, *p* < 0.001, ES = 2.09) compared with those in the UP group (3.02 ± 1.93%, *p* < 0.001, ES = 1.54). A 3.08% increase in time to perform 10-yd (ES = 0.69, *p* = 0.001) was also noted. Our results show an effect of periodization on time variations at 10-yd, with a significant improvement of 4.71 ± 4.11% (ES = 1.10) for athletes in the BP group vs. a non-significant gain of 1.26 ± 4.69% (ES = 0.28) for players in the UP group. The players of the BP group significantly improved their 20-yd time by 1.48 ± 2.53% (3.13 ± 0.25 vs. 3.08 ± 0.28s, *p* = 0.039, ES = 0.59) whereas a 0.35 ± 2.63% drop in performance was found in players of the UP group, for whom the time to 20-yd went from 3.00 ± 0.19 to 3.01 ± 0.20s (*p* = 0.670, ES = −0.13).

**Table 3:** Effect of training modality on sprint parameters.

Variables	Group	Pre	Post	Δ%	*p* (training)	*p* (group)
40-yd (s)	Block (*n* = 15)	5.55 ± 0.53	5.30 ± 0.52	−4.45 ± 2.06	<0.001*	0.079
	undulating (*n* = 12)	5.32 ± 0.35	5.16 ± 0.36	−3.02 ± 1.93	<0.001*	
	Total (*n* = 27)	5.45 ± 0.46	5.24 ± 0.45	−3.72 ± 2.11	<0.001*	
20-yd (s)	Block (*n* = 15)	3.13 ± 0.29	3.08 ± 0.28	−1.48 ± 2.53	0.039*	0.078
	undulating (*n* = 12)	3.00 ± 0.19	3.01 ± 0.20	0.35 ± 2.63	0.670	
	Total (*n* = 27)	3.07 ± 0.25	3.05 ± 0.24	−0.56 ± 2.69	0.186	
10-yd (s)	Block (*n* = 15)	1.86 ± 0.17	1.77 ± 0.17	−4.71 ± 4.11	<0.001*	0.052
	undulating (*n* = 12)	1.76 ± 0.11	1.74 ± 0.13	−1.26 ± 4.69	0.356	
	Total (*n* = 27)	1.81 ± 0.15	1.76 ± 0.15	−3.08 ± 4.57	0.001*	
V_max_ (m.s ^−1^ )	Block (*n* = 15)	7.70 ± 0.89	8.24 ± 0.86	7.30 ± 6.63	<0.001*	0.347
	undulating (*n* = 12)	8.10 ± 0.57	8.46 ± 0.62	4.54 ± 4.10	0.002*	
	Total (*n* = 27)	7.88 ± 0.78	8.34 ± 0.76	6.13 ± 5.62	<0.001*	
VH_0_ (m.s^−1^)	Block (*n* = 15)	8.23 ± 0.95	8.50 ± 0.93	3.42 ± 4.31	0.009*	0.236
	undulating (*n* = 12)	8.52 ± 0.64	8.64 ± 0.68	1.48 ± 3.88	0.214	
	Total (*n* = 27)	8.36 ± 0.83	8.56 ± 0.82	2.68 ± 4.14	0.004*	
FH_0_rel (*N*.kg ^−1^ )	Block (*n* = 15)	7.44 ± 0.71	7.15 ± 0.88	−3.81 ± 7.96	0.069	0.269
	undulating (*n* = 12)	7.47 ± 0.69	7.47 ± 0.94	0.10 ± 10.03	1.00	
	Total (*n* = 27)	7.45 ± 0.69	7.29 ± 0.91	−2.20 ± 8.84	0.238	
PH_max_rel (W.kg ^−1^ )	Block (*n* = 15)	15.24 ± 2.80	15.23 ± 3.14	−0.31 ± 7.21	0.964	0.637
	undulating (*n* = 12)	15.82 ± 2.29	16.06 ± 2.98	1.18 ± 9.03	0.599	
	Total (*n* = 27)	15.50 ± 2.55	15.60 ± 3.04	0.36 ± 7.79	0.699	

*-*yd, yards; V_max_, maximum speed; VH_0_ is the theoretical maximum speed up to which the athlete is still capable of producing positive horizontal force; FH_0_rel is the theoretical maximum horizontal force an athlete can produce on the ground relative to body mass; PH_max_rel: the maximum power an athlete can develop in a horizontal direction relative to body mass.

* Significant difference *p* < 0.05.

Our results showed a significant improvement in V_max_ of 6.13 ± 5.62% (7.88 ± 0.78 vs. 8.34 ± 0.76 m.s^−1^, *p* < 0.001, ES = −1.21) and VH_0_ of 2.68 ± 4.14% (8.36 ± 0.83 vs. 8.56 ± 0.82 m.s^−1^, *p* = 0.004, ES = −0.61) without a group effect. A greater gain was noted for players in the BP group compared to those in the UP group for V_max_ (7.30 ± 6.63 vs. 4.54 ± 4.10, *p* = 0.002, ES = 1.30). VH_0_ increased significantly only in the BP group (3.42 ± 4.31%, *p* = 0.009, ES = −0.78). Our results show a relationship between variation in time over 40-yd and variation in V_max_ (r = −0.43, *p* = 0.025) and VH_0_ (r = −0.4, *p* = 0.039). No significant difference was observed on the mechanical variable of PH_max_rel (0.36 ± 7.79%, *p* = 0.699, ES = −0.08). No group effect was observed between the two training modalities (−0.31 ± 7.21 vs. 1.18 ± 9.03%, *p* = 0.964, ES = −0.15, for BP and UP, respectively). Our results show a decrease in FH_0_rel of 2.20 ± 8.84% (7.45 ± 0.69 vs. 7.29 ± 0.91 N.kg^−1^, *p* = 0.238, ES = 0.23). This reduction was visible in athletes in the PB group (−3.81 ± 7.96%, *p* = 0.069, ES = 0.54) but not in the UP group (0.10 ± 10.03%, *p* = 1.00, ES = 0.00).

## Discussion

The aim of this study was to compare the effects of two training periodization modalities (block vs. undulating periodizations) on 40-yard performance. Our main results show an improvement in time and V_max_ at 40-yd with training and with no effect of the type of periodization. However, block periodization (BP) appears to exert more greater effects than undulating periodization (UP) on the progression of V_max_ and 40-yd sprint time. A cross-training periodization effect is observed on times at 10-yd and 20-yd and on the value of VH_0_, but neither UP nor BP have a significant effect on FH_0_rel and PH_max_rel values. First, we will discuss anthropometric characteristics during the training period.

Our results show no significant difference between BM, fractions of lean body mass and fat mass values between our two groups before and after training. BM values increased during the training period. Our results are similar to those of Painter et al. (19), who also showed an increase in BM with no effect of training modality in highly trained athletes. In our study, the increase in BM is explained by a significant gain in BM for the UP group and not significant for the BP group. Gavanda et al. (14) have previously shown a stronger effect of training period on BM in athletes undergoing UP compared to their counterparts undergoing BP. This variation in BM observed by Gavanda et al. (14) was associated with a decrease in fat mass and an increase in lean body mass in their two groups of AF players. However, these changes were more marked in athletes in the UP group after the four four-week mesocycles of training (a total of sixteen weeks of training). The variations in fat mass and lean body mass observed by Gavanda et al. (14) could explain our results. Indeed, we observed that the increase in body mass was contemporaneous with a trend towards a decrease in fat mass and an increase in lean body mass (+2.1 vs. + 0.4 kg) more marked in our athletes in the UP group than in the BP group. In our study, the improvement in BM in the UP group could also be explained by a training-induced change in bone mass. Indeed, Almstedt et al. (48) observed an increase in bone density from 2.2% to 7.0% following 24 weeks of undulatory strength training.

No correlation was found between variations in body mass or lean body mass and time over 40-yd. These results are in agreement with those of Abe et al. (49), who showed no relationship between lean body mass and 100-m performance. In our study, we observed a 3.72% improvement in 40-yd performance. This observation can be explained by a significant reduction in running time of −4.45% for the BP group (ES = 2.09) and −3.02% for the UP group (ES = 1.54). Previously Gavanda et al. (14) had shown a significant effect of the same types of training (BP and UP) on sprint performance, with a small effect size (ES = 0.2 and 0.32 for BP and UP, respectively). These differences in ES between the two studies may be explained by the use of a different training methodology. In the study of Gavanda et al. (14), athletes performed a non-specific training method, i.e., strength training without specific sprint work. In our study, both groups performed muscle-strengthening sessions combined with sprint work, with or without weights. It has been shown by Rumpf et al. (44) that the use of a specific training method improved sprint performance more than a more general method. Furthermore, the differences observed between our results and those of Gavanda et al. (14) could also be explained by different development objectives. Indeed, in their study, Gavanda et al. (14) focused on the development of muscular endurance, hypertrophy and muscular strength, whereas our protocol was based on the development of strength, power and maximum speed. These lines of development could also explain our observation of an improvement in time over 10-yd. Indeed, as shown by Lockie et al. (50), weighted sprinting or heavy-load strength training work induced better speed gains over 10-m in sprinting, i.e., around 10.9-yd.

However, our results show an effect of periodization on time variations at 10-yd, with a significant improvement of 4.71 ± 4.11% (ES = 1.10) for athletes in the BP group vs. a non-significant gain of 1.26 ± 4.69% (ES = 0.28) for players in the UP group. To our knowledge, no study has investigated the effects of periodization (BP vs. UP) on sprint performance over a distance of less than 20-m or 20-yd. Our results show an effect of periodization on time variations at 20-yd with training. Thus, athletes in the BP group significantly improved their 20-yd time by 1.48 ± 2.53% (3.13 ± 0.25 vs. 3.08 ± 0.28s, *p* = 0.039, ES = 0.59). Conversely, our results show a 0.35 ± 2.63% drop in performance for players in the UP group, for whom the time to 20-yd went from 3.00 ± 0.19 to 3.01 ± 0.20s (*p* = 0.670, ES = −0.13). These observations are similar to the results of the study by Sabido et al. (20) who reported a non-significant decrease in time to 20-m in nine adolescent handball players (15.3 ± 0.5 years old) after eight weeks of BP. This more pronounced trend towards a decrease in 20-m time was accompanied by a greater improvement in horizontal jump performance in the young players following BP compared with the UP group (20). Previously Brechue et al. (51) had shown a significant relationship between average speed over 20-yd and long jump performance (i.e., BJ). Two recent studies showed that BP had the best effects on strength in volleyball players (52) and in young hockey players (53). In our study, the variation in performance on sprint times would seem to indicate that BP would be more effective than UP in improving the acceleration capacity of young American football players. BP appears to be more effective than UP for sprint performance. In our study, improvements in 10-yd, 20-yd, and 40-yd sprint times were greater for BP than for UP. These findings are supported by Sabido et al., who observed a moderate effect size (ES = −0.51) improvement in the 20-m sprint time for BP but no significant effect. Additionally, both groups showed significant improvements in jump performance, though the gains were more pronounced for BP (9.7% vs. 4.0% for BP and UP, respectively). However, in the study by Gavanda et al. (14), 40-yd sprint performance significantly improved, but no difference was found between groups. The discrepancy between these studies may stem from differences in training programs. Sabido et al. implemented a program focused on neural development (power and explosive strength), whereas Gavanda et al. (14) designed their training around muscular development (endurance and hypertrophy). Together, the results of these previous studies suggest that responses to strength and sprint training are sensitive to periodization types. To better understand the difference between the BP and UP effects on sprint performance, we looked at the effects of periodization on the responses of mechanical variables to training.

Our results show a 6.13% improvement in maximum velocity (V_max_) values and a 2.68% improvement in theoretical maximum horizontal velocity (VH_0_) with training. This result is in agreement with the work of Slawinski et al. (9), who showed a relationship between improved sprint performance and changes in V_max_ and VH_0_ values with training. As with the V_max_ variable, changes in VH_0_ were more marked for players in the BP condition than their counterparts in the UP group, with a significant improvement in VH_0_ with training only for the BP group. The periodization effect could explain this observation. According to observations by Hicks et al. (40), sprint sessions with weighted sled improved times over 10-yd and 20-yd and the value of VH_0_, as well as V_max_. The improvement in V_max_ would be due in part to a better index of technical application of ground force (DRF) following training sessions with weighted and assisted sprints (8, 54). According to Morin et al. (8), the value of V_max_ is also correlated with those of VH_0_ and PH_max_rel. In our study, the ten weeks of training induced a non-significant gain in PH_max_rel of 0.36%. This was due to a 2.20% drop in FH_0_, which was not offset by the 2.68% increase in VH_0_. Indeed, according to equation 4, PH_max_rel is the product of VH_0_ and FH_0_ per unit body mass. Our results show an effect of periodization on variations in FH_0_. Indeed, athletes in the BP group showed a 3.81 ± 7.96% drop in FH_0_, while players in the UP group maintained their strength levels (+0.10 ± 10.03%). The observed effect of periodization on FH_0_ and PH_max_rel values could be explained by a different distribution of maximum strength development sessions between BP and UP. Gains in FH_0_rel have been reported in athletes following sixteen training sessions with weighted sled (24). Cahill et al. and Lathi et al. (21, 23) observed significant increases in strength following weighted sled sessions, whereas FH_0_rel values were maintained or decreased in athletes whose training was based on repetition of unresisted or assisted sprints (21, 23). In 23 young sprinters, Martínez-Valencia et al. (55) previously showed that use of a load up to 20% of body mass could provide a training stimulus in young sprinters to improve the peak value of the rate of force development (RFD) during the sprint start, and thus, early acceleration. The RFD values has been considered a factor that influences performance in explosive playing actions (56, 57). RFD determines the force that can be generated in the early phase of muscle contraction (55). Ishøi et al. (56) observed significant relationship RFD, FH_0_, P_max_ and sprint time (0- to 5-m and 0- to 30-m). Many elite sprinters follow a combined program consisting of resistance training and sprint training to increase RFD values (58). Batra et al. (58) showed that BP training was effective in raising the physical capabilities directly transferable to sprinting. Comparative studies in American Football players and other strength athletes showed a greater adaptations after BP compared to UP (18). Nonetheless, players of BP group did not improve or maintain their FH_0_rel value. This finding could be explained by the distribution of the maximum speed development based on un-resisted sprint sessions. In previous studies, sprint evaluation was conducted at the end of the training period (21, 23). In our study, only three sessions were based on the development of FH_0_rel which were distributed differently according to training modalities. The periodization in our study for the BP group did not allow us to carry out assessments following the three heavy sled training sessions, but only nine weeks later (four weeks of power, three weeks of speed and two weeks of training relief). It has already been reported that un-resisted sprint training did not improve acceleration performance in untrained and trained subjects (59, 60). Thus, these many weeks without weighted sprints with heavy loads could perhaps explain the decrease in FH_0_rel value of BP group players.

On the other hand, while BP appears to be more effective in developing variable associated with sprinting performance, a minimum interval between sessions is necessary to optimize the effects of training. Hartmann et al. (18) previously reported that a minimum recovery period of 72 h was required to restore high levels of performance in American football players and others. Thus, the integration of strength training into in-season conditioning depends on the duration of the competition period, the frequency of contests, and the proportion of the conditioning program (18). In their meta-analyse, Hartmann et al. (18) suggested that two weekly strength sessions are optimal for maximizing training effects on mechanical variables and sprinting performance. Rønnestad et al. (61) also proposed a weekly training session targeting a non-prioritized physical quality to improve and maintenance over time strength and power qualities (62). The schedule in team sports is increasingly demanding, leaving little time to develop the physical qualities necessary for performance (63). For this reason, it is beneficial for strength and conditioning coaches to focus on BP during the off-season, as it has proven effective in improving key variables within a short periodization (61). To maintain these improvements, UP should be employed, taking into account the frequency of competitions during the week, which can be quite frequent in some sports (64).

Explosive playing actions (i.e., accelerations, sprinting, rapid changes of direction, and powerful movements such as kicking, tackling or striking) are critical for performance in team sports, as they involve high-intensity, short-duration movements that contribute to success in competition (65–68). Precautions must be taken into account when applying our results to other populations. Sled towing is a highly beneficial exercise for enhancing force output and improving the acceleration phase of sprinting (55). Sled towing could be integrated into the early stages of a sprint training program to build strength and explosive power. According to a study comparing BP and UP among trained field athletes, BP appears to be more favorable for maximizing specific qualities (strength, hypertrophy) during the off-season or pre-season in male and female athletes (53, 69–71). BP may also provide more favorable results in terms of resting hormone levels and injury reduction (72). Thus, BP may be used in the off-season or pre-season to allow for optimal recovery and adaptation. BP is also recommended for untrained individuals as it helps to build a solid strength foundation in male and female (73). However, UP might be more advantageous for athletes who need frequent variations in training intensity. For track and field athletes, UP would be more appropriate during the competitive season to prevent stagnation and maintain consistent progress (70). In untrained and elite female handball players, it was shown that BP promoted maximal strength development, while UP was more beneficial for maintaining and improving endurance and agility during the competition period (74, 75). As a result, periodization should be tailored to the athlete's specific goals (e.g., sprinting, muscular strength, or injury prevention), experience level, and the phase of the season. BP is more beneficial for specific strength development and recovery, while DUP is better suited for maintaining performance and preventing stagnation. Finally, coaches might consider integrating sled towing and resistance training during the off-season in order to optimize the peak force, RFD, and sprint acceleration performance.

## Conclusion

Both training modalities showed a significant improvement in performance at 40-yd. It would appear that UP is more suitable for increasing muscle mass and maintaining the mechanical variables FH_0_rel and PH_max_rel. Conversely, the focus on physical qualities during BP would enable these to be developed. As a result, BP would be more suitable for young players who are inexperienced in training. Depending on the sporting calendar, the use of BP, whose aim is to improve the mechanical variables associated with sprint performance, would be judicious in the inter- or pre-season. The aim is to maintain these variables with UP during the season.

## Data Availability

The datasets presented in this study can be found in online repositories. The names of the repository/repositories and accession number(s) can be found in the article/Supplementary Material.
